# *Clostridium difficile* has a single sortase, SrtB, that can be inhibited by small-molecule inhibitors

**DOI:** 10.1186/s12866-014-0219-1

**Published:** 2014-08-31

**Authors:** Elizabeth H Donahue, Lisa F Dawson, Esmeralda Valiente, Stuart Firth-Clark, Meriel R Major, Eddy Littler, Trevor R Perrior, Brendan W Wren

**Affiliations:** Pathogen Molecular Biology Department, London School of Hygiene and Tropical Medicine, Keppel Street, London, WC1E 7HT UK; Domainex Ltd, 162 Cambridge Science Park, Milton Road, Cambridge, CB4 0GH UK

**Keywords:** *Clostridium difficile*, Sortase, Cysteine protease, Fluorescence resonance energy transfer (FRET), Enzyme kinetics, Enzyme inhibitors

## Abstract

**Background:**

Bacterial sortases are transpeptidases that covalently anchor surface proteins to the peptidoglycan of the Gram-positive cell wall. Sortase protein anchoring is mediated by a conserved cell wall sorting signal on the anchored protein, comprising of a C-terminal recognition sequence containing an “LPXTG-like” motif, followed by a hydrophobic domain and a positively charged tail.

**Results:**

We report that *Clostridium difficile* strain 630 encodes a single sortase (SrtB). A FRET-based assay was used to confirm that recombinant SrtB catalyzes the cleavage of fluorescently labelled peptides containing (S/P)PXTG motifs. Strain 630 encodes seven predicted cell wall proteins with the (S/P)PXTG sorting motif, four of which are conserved across all five *C. difficile* lineages and include potential adhesins and cell wall hydrolases. Replacement of the predicted catalytic cysteine residue at position 209 with alanine abolishes SrtB activity, as does addition of the cysteine protease inhibitor MTSET to the reaction. Mass spectrometry reveals the cleavage site to be between the threonine and glycine residues of the (S/P)PXTG peptide. Small-molecule inhibitors identified through an *in silico* screen inhibit SrtB enzymatic activity to a greater degree than MTSET.

**Conclusions:**

These results demonstrate for the first time that *C. difficile* encodes a single sortase enzyme, which cleaves motifs containing (S/P)PXTG *in-vitro*. The activity of the sortase can be inhibited by mutation of a cysteine residue in the predicted active site and by small-molecule inhibitors.

**Electronic supplementary material:**

The online version of this article (doi:10.1186/s12866-014-0219-1) contains supplementary material, which is available to authorized users.

## Background

Sortases are membrane-bound cysteine transpeptidases that anchor surface proteins to the peptidoglycan cell wall in Gram-positive bacteria. Surface proteins anchored via sortases are often essential virulence factors important in colonization and invasion, evasion of the host immune system, and nutrient acquisition. The sorting process is mediated by a conserved C-terminal cell wall sorting signal on the anchored protein, comprised of a C-terminal recognition sequence (often LPXTG, where X is any amino acid), followed closely by a hydrophobic transmembrane domain and a positively charged tail [1]. A conserved catalytic cysteine residue of the sortase cleaves the LPXTG motif of the polypeptide between the threonine and glycine residues and covalently attaches the protein to the peptidoglycan [2–6].

There are six described sortase families, A-F, that share amino acid similarity [7]. All catalyze similar transpeptidation reactions, but recognize different substrate motifs and serve different functions within the cell. Class A sortases (SrtA), such as the prototypical *Staphylococcus aureus* Sortase A (SaSrtA), are considered housekeeping sortases as they are capable of anchoring many functionally distinct proteins to the cell wall. SaSrtA, which recognizes an LPXTG motif, is responsible for anchoring a variety of surface proteins involved in adherence and immune response evasion, and is essential for virulence in animal models [8,9]. SrtA orthologues have been found in the genomes of almost all Gram-positive bacteria [8,10–16]. Class B sortases are functionally different from class A in their substrate specificity. In *S. aureus* and *B. anthracis*, the sortase B gene (*srtB*) is part of an iron-regulated locus *isd* (**i**ron-responsive **s**urface **d**eterminant) responsible for heme-iron transport, and anchors the iron transporter protein, IsdC, by recognizing an NPQTN motif [17,18]. Though mutating *srtB* has no effect on establishing infection, SaSrtB is required for persistence of the bacterium in mice [17].

*Clostridium difficile*, an anaerobic Gram-positive, spore-forming bacillus, is the leading cause of hospital-acquired infectious diarrhea in North America and Europe. Infection with *C. difficile* can result in a range of clinical presentations, from mild self-limiting diarrhea to the life-threatening pseudomembranous colitis (PMC), known collectively as *C. difficile* infection (CDI) [19]. MLST studies have identified that the *C. difficile* population structure forms at least five distinct lineages that are all associated with CDI [20–22]. Complications of severe CDI can lead to toxic megacolon, bowel perforation, sepsis and death in up to 25% of cases [23]. Broad-spectrum antibiotic usage is the greatest risk factor for development of CDI due to the consequent disruption of the intestinal microflora. Treatment of CDI with metronidazole and vancomycin can exacerbate the problem by continuing to disrupt the intestinal microflora. This leaves the patient susceptible to relapse or re-infection. Approximately one third of patients experience CDI relapse following treatment, and those who relapse have a greater risk of succumbing to the infection [23]. A current imperative is the development of therapies that selectively target *C. difficile*, whilst leaving the intestinal microflora intact.

The *C. difficile* reference strain 630 encodes a single predicted sortase, CD630_27180, which has high amino-acid similarity with SrtB of *S. aureus* and *B. anthracis* [24]. A second sortase encoded within the genome is interrupted by a stop codon prior to the catalytic cysteine and is considered a pseudogene. Thus, in contrast to other Gram-positive bacteria, *C. difficile* appears to have only a single functional sortase. As such, a compound that inhibits the activity of *C. difficile* sortase could target the pathogen without disrupting the numerous Gram-negative bacteria that make up the intestinal flora.

In this study, we demonstrate that the predicted sortase encoded by CD630_27180 recognizes and cleaves an (S/P)PXTG motif between the threonine and glycine residues. The cleavage of this motif is dependent on the conserved cysteine residue at position 209 in the predicted active site of the sortase. We have also identified seven putative sortase substrates, all of which contain the (S/P)PXTG motif. These substrates are conserved among the five *C. difficile* lineages and include potential adhesins, a 5’ nucleotidase, and cell wall hydrolases. Furthermore, we identified a number of small-molecule inhibitors by means of an *in silico* screen that inhibit the activity of the *C. difficile* SrtB.

## Results

### Conservation of the catalytically active residues of sortase

**Figure 1:**
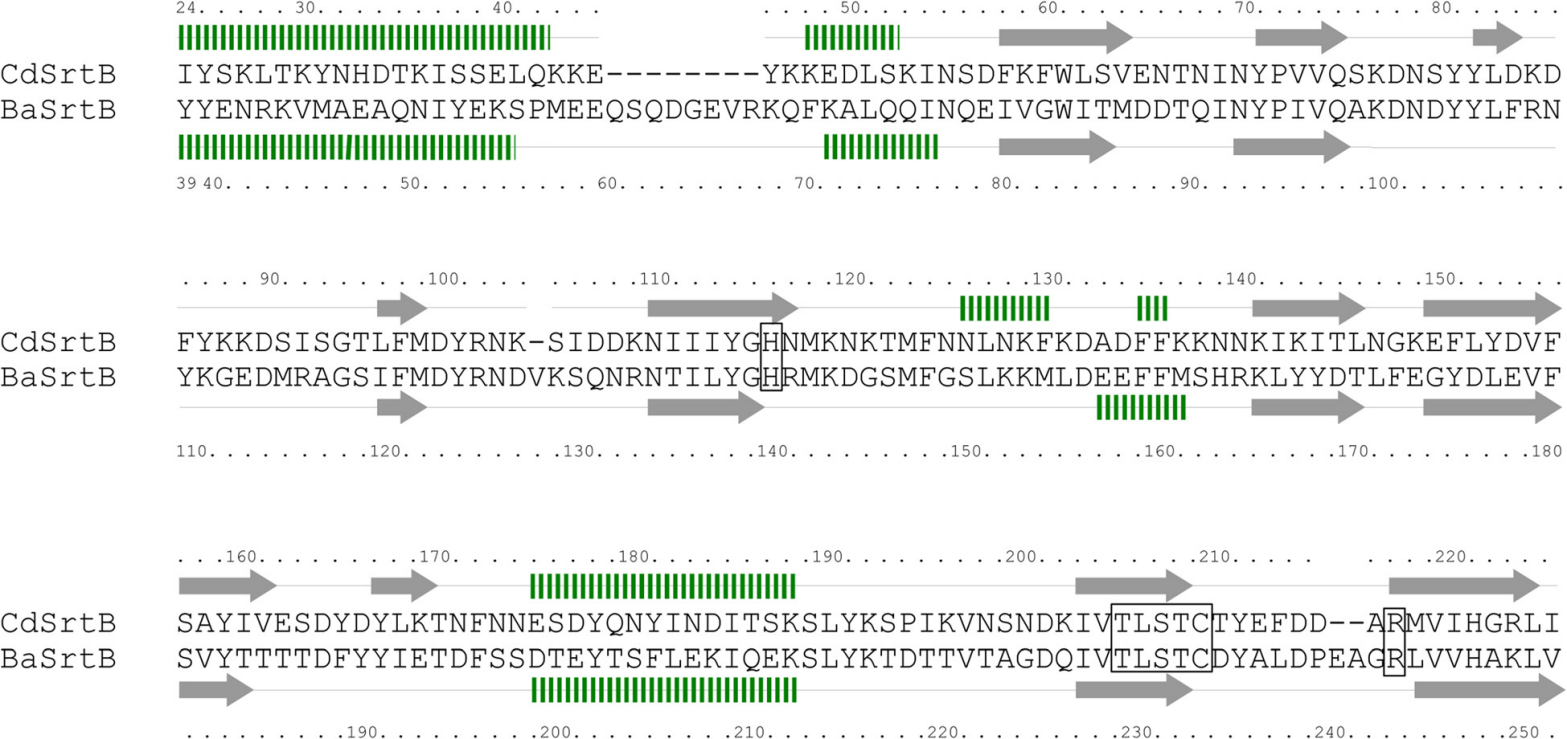
**Predicted** ***C. difficile*** **SrtB secondary structure** ***.*** A structural alignment between the known crystal structure of BaSrtB [28] and the predicted structure of *C. difficile* SrtB using the Phyre2 Protein Fold Recognition Server suggests a high degree of structural conservation. Top: *C. difficile* SrtB predicted secondary structure and sequence. Bottom: BaSrtB sequence and known structure. Arrows indicate beta sheets, and striped rectangles indicate alpha helixes. Amino acid positions relative to start position are indicated. The sortase active site signature sequence TLXTC is boxed, as are the conserved essential histidine and arginine residues.

The genome sequence of *C. difficile* strain 630 previously indicated the presence of a single copy of a sortase-like protein, encoded by *CD630_27180*, based on the presence of the sortase catalytic motif TLXTC [24]. We performed BLAST searches (BlastP) to reveal the protein encoded by *CD630_27180* shares 32% and 34% amino acid identity with SrtB from *S. aureus* (SaSrtB) and *B. anthracis* (BaSrtB), respectively. In addition to the TLXTC active site, the catalytically essential histidine (His120 in SaSrtA) and arginine (R197 in SaSrtA) residues [3,25,26] are conserved in the *C. difficile* SrtB. A structural prediction analysis of SrtB was performed using Phyre2 Protein Fold Recognition Server (http://www.sbg.bio.ic.ac.uk/phyre2/html/page.cgi?id=index) [27], and the resulting alignment suggests a high level of conservation between the predicted secondary structure of SrtB and the known crystal structure of the BaSrtB [28] (Figure 1). Expression of *C. difficile* SrtB was analysed *in vitro* using RT-PCR analysis on strain 630, which confirmed that *CD2718* is actively transcribed during early exponential, late exponential and stationary phases (Additional file 1: Figure S1).

The *C. difficile* population structure forms at least five distinct clonal lineages that are all associated with human infection [20–22]. To determine whether SrtB is conserved between *C. difficile* strains, representatives for each of the five distinct clades were chosen for analysis based on the availability of a fully annotated sequence: *C. difficile* strains 630 for Clade 1, R20291 and CD196 (RT027) for Clade 2 [29], M68 and CF5 (RT017) for Clade 3 [20], CD305 (RT023) for Clade 4 (unpublished, WTSI), and M120 (RT078) for Clade 5 [20]. BLAST searches of these representative strains show that *srtB* is conserved across all five *C. difficile* lineages. A second sortase-like gene in the 630 genome, classified as a pseudogene because of an in frame stop codon prior to the catalytic cysteine, is absent from the other four *C. difficile* lineages.

### Bioinformatic prediction of sortase substrates

A bioinformatics approach was used for the preliminary identification of sortase substrate proteins in *C. difficile* strain 630. The predicted recognition sequence for CD630_27180 has been proposed to be (S/P)PXTG by Pallen *et al.* [11], and recently to also include the sequence NVQTG, found in the surface- associated collagen binding protein CbpA, by Tulli *et al.* [30]. Putative proteins were screened for the patterns (S/P)PXTG and NVQTG at the C-terminus [11,30]. Putative candidates were then assessed for the known features of a sortase substrate: a predicted N-terminal signal peptide sequence, and a cell wall sorting signal comprising of a potential transmembrane domain following the sortase recognition sequence, and at least two consecutive basic residues (arginine or lysine) at the C-terminus [31–33].

Eight proteins satisfied our definition of a sortase substrate in strain 630 (Table 1). The newly described *C. difficile* collagen binding protein A, CbpA, is the only protein containing the proposed NVQTG motif [30]. The remaining proteins contained one of four observed variations of the (S/P)PXTG motif: SPKTG, PPKTG, and SPSTG and SPQTG. These predicted *C. difficile* sortase substrates are a diverse range of surface proteins that include putative cell wall hydrolases, putative adhesins, a collagen-binding protein, and a 5’ nucleotidase/phosphoesterase (Table 1). Transcriptional analysis performed by RT-PCR confirmed that all eight predicted substrate proteins are transcribed during growth *in vitro* (Additional file 1: Figure S1B-I). The eight predicted substrates are transcribed during all three growth phases examined, with the exception of CD630_25370 and CD630_32460, which do not appear to be transcribed during stationary phase. Four of these putative substrates are conserved across all five *C. difficile* lineages: CD630_01830, CD630_25370, CD630_27680, and CD630_28310.

**Table 1:** **Identification of putative** ***C. difficile*** **SrtB substrates in strain 630**

**Protein**	**Function**	**C-terminal sorting signal**
CD630_01830	Putative cell wall hydrolase	MIH**SPSTG**KT***V***S***V***TS***I***NSS***YY***TAR***FV***T***A*** *KR*IL
CD630_03860	Putative cell surface protein, collagen binding protein	PSD**SPKTG**DNTN***LY***G***LLALLL***TSG***A***G***LA***G***IFFY*** *KRRK*M*KK*S
CD630_25370	Putative membrane-associated 5'-nucleotidase/phosphoesterase	KEK**SPKTG**D***L***G***F***SNS***IIIFIV***SST***LI***C***LL***N***F***NQ*K*EL*K*D*KK*S*K*
CD630_27680	Putative cell-wall hydrolase	FIH**SPQTG**D***VV***K***V***TS***MA***PGTN***YA*** *RR*LITAT*R*VLQ
CD630_28310	Putative adhesion, collagen binding protein	PPV**PPKTG**DSTT***II***GE***ILLVI***G***AIV***G***LIVL*** *RR*N*K*NTN
CD630_31450	Collagen binding protein, CbpA	VGQ**NVQTG**DQSN***IML***D***LALMFI***S***LFFLI*** *K*NLTN*K*YL*RRK*
CD630_32460	Putative surface protein	IVK**SPKTG**DETQ***LM***S***YVFI***S***VIAI***CG***LAY***QC*K*I*KR*N
CD630_33920	Putative cell surface protein, collagen binding protein	PSD**SPKTG**DSTN***LMAFIVMLLV***SGGG***LA***GT***YLY*** *KRRK*M*KK*S

Bold = predicted sortase recognition sequence.

Bold and Italic = hydrophobic residues.

Italics only = positively charged residues.

### Purified *C. difficile* SrtB cleaves (S/P)PXTG peptides

To determine whether *C. difficile* SrtB cleaves putative substrates at the predicted motifs, FRET peptides were designed based on the variations observed in the predicted (S/P)PXTG motif (Table 2). Two residues upstream of the motif were included, and two glycine residues were incorporated downstream, as this has been previously shown to improve sortase cleavage efficiency *in vitro* [34]. Fluorescence of the Edans fluorophore within the peptides is blocked when in close proximity to the fluorescent quencher Dabcyl [35]. When the peptide is cleaved, the Edans fluorophore is separated from Dabcyl, and a fluorescent signal is observed.

**Table 2:** **FRET peptide details**

Peptide sequence*	**Description**
*d-*IHSPSTGGG*-e*	Based on CD0183 sequence
*d-*IHGSSTPGG*-e*	Control for above peptide
*d-*SDSPKTGGG*-e*	Based on CD0386, CD3392 sequence
*d-*SDGSKTPGG*-e*	Control for above peptide
*d-*IHSPQTGGG*-e*	Based on CD2768 sequence
*d-*IHGSQTPGG*-e*	Control for above peptide
*d-*PVPPKTGGG*-e*	Based on CD2831 sequence
*d-*PVGPKTPGG*-e*	Control for above peptide
*d-*GQNVQTGGG*-e*	Based on CbpA sequence
*d-*QALPETGGG*-e*	SaSrtA peptide
*d-*NPQTN*-e*	SaSrtB peptide
*d-*IHSPSTGKT*-e*	Based on CD0183 sequence
*d-*SDSPKTGDN*-e*	Based on CD0386 sequence
*d-*IHSPQTGDV*-e*	Based on CD2768 sequence
*d-*PVPPKTGDS*-e*	Based on CD2831 sequence

*Where *d* is Dabcyl (4-([4-(dimethylamino)phenyl]azo)-benzoyl) and *e* is Edans (5-((2-Aminoethyl)amino)naphthalene-1-sulfonic acid).

**Figure 2:**
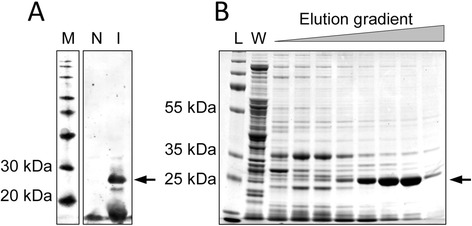
**Expression and purification of SrtB**_Δ**N26**_ **.** *E. coli* NiCo21(DE3) expressing SrtB_ΔN26_, in which the N-terminal membrane anchor has been replaced with a six-histidine tag, were lysed by sonication and cleared lysates purified by nickel affinity chromatography. **A**. Anti-his western testing for expression of SrtB_ΔN26_. Lane M: molecular mass marker, N: whole cell lysate of non-induced culture, I: whole cell lysate of culture induced with 1 mM IPTG. **B**. Coomassie-stained SDS-PAGE analysis of SrtB_ΔN26_ purification over an imidazole gradient. Lane L: molecular mass marker, W: column wash, imidazole gradient indicated by grey triangle, arrows indicate the SrtB_ΔN26_ protein_._

**Figure 3:**
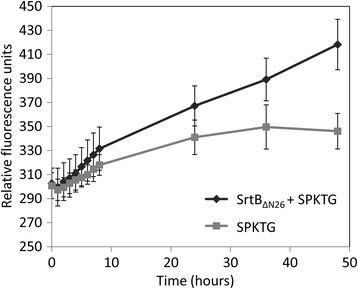
**Cleavage of SPKTG peptide by recombinant SrtB**_Δ**N26**_ **.** Purified recombinant SrtB_ΔN26_ was incubated with a FRET peptide containing the SPKTG motif and fluorescence measured every hour for the first eight hours, and also at 24 h, 36 h, and 48 h. An increase in relative fluorescence units (arbitrary units) was observed, compared with the peptide incubated alone, indicating that SrtB_ΔN26_ mediated cleavage of the SPKTG peptide occurred over a period of 48 hours. Error bars represent the standard error of the mean.

**Figure 4:**
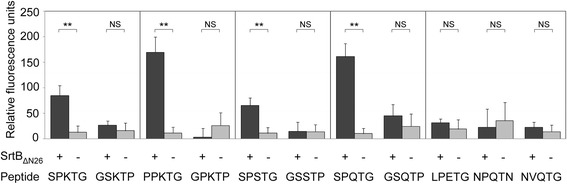
**SrtB**_Δ**N26**_ **substrate specificity.** Purified recombinant SrtB_ΔN26_ protein was incubated with a range of peptide sequences to investigate its substrate specificity. The motifs SPKTG, PPKTG, SPSTG and SPQTG were all recognized and cleaved following incubation with SrtB_ΔN26_. The scrambled peptide sequences GSKTP, GPKTG, GSSTP, and GSQTP serve as controls for the cleavage specificity of SrtB_ΔN26_. The sequences LPETG and NPQTN, corresponding to the motifs recognized by *S. aureus* sortase A and B, respectively, do not appear to be substrates for SrtB_ΔN26_. SrtB_ΔN26_ also failed to cleave the proposed sorting signal NVQTG from recently characterized collagen binding protein, CbpA. Bars indicate the mean, and error bars represent the standard error (**corresponds to *p* < 0.01).

The N-terminal transmembrane domain of *C. difficile* SrtB (residues 2–25) was replaced with a six-histidine tag (SrtB_ΔN26_) to improve soluble protein yield. SrtB_ΔN26_ was expressed in *E. coli* NiCo21(DE3) and purified by nickel affinity chromatography from cleared lysates (Figure 2). Purified SrtB_ΔN26_ was then incubated with a FRET peptide containing the SPKTG sequence. An increase in fluorescence was observed over time, indicating that cleavage of the SPKTG peptide occurred in the presence of SrtB_ΔN26_ over 48 hours (Figure 3). In addition to the SPKTG motif, SrtB_ΔN26_ also cleaved peptides containing the predicted substrate sequences PPKTG, SPSTG, and SPQTG (Figure 4). SrtB_ΔN26_ failed to cleave the scrambled peptide sequences GSKTP, GPKTP, GSSTP and GSQTP (Figure 4). Interestingly, SrtB_ΔN26_ failed to cleave peptides containing the LPETG and NPQTN motifs of SaSrtA and SaSrtB, respectively, and also failed to cleave the proposed sortase recognition motif NVQTG found in the *C. difficile* collagen binding protein, CbpA [30] (Figure 4).

### Analysis of FRET reaction

**Figure 5:**
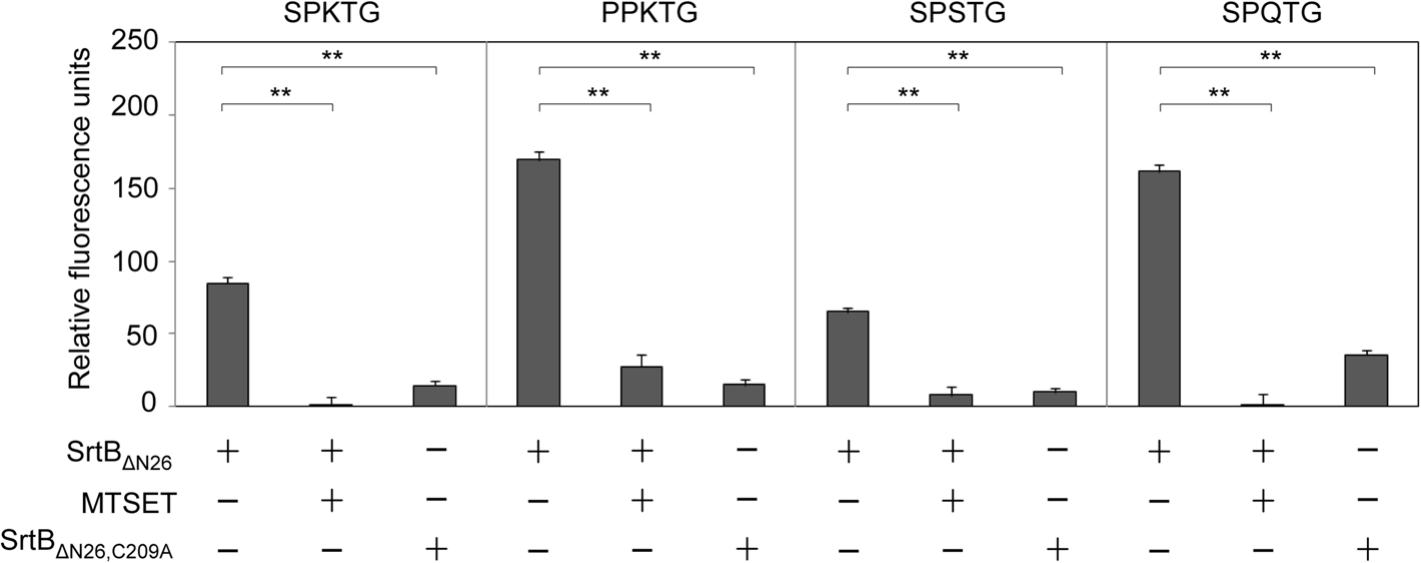
**SrtB**_Δ**N26**_ **activity requires a cysteine residue at position 209.** To determine if SrtB_ΔN26_ activity depended on the cysteine residue at position 209, a C209A substitution was made to create SrtB_ΔN26,C209A_. This enzyme was inactive against the FRET peptides when compared with SrtB_ΔN26_. Addition at 5 mM of the cysteine protease inhibitor, MTSET, to the reaction also eliminates activity (**corresponds to *p* < 0.01).

To investigate the importance of the cysteine residue in the proposed active site of *C. difficile* SrtB, site-directed mutagenesis was used to replace the cysteine residue at position 209 with an alanine. When the resulting mutant protein SrtB_ΔN26,C209A_ was incubated with the FRET peptides, the fluorescent signal fell below the limits of detection (Figure 5), indicating that the cysteine residue at position 209 was essential for the activity of the *C. difficile* SrtB. Cleavage in the FRET-based assay was also inhibited by the addition of MTSET (Figure 5), a known cysteine protease inhibitor and inhibitor of sortase function in *S. aureus* [36,37] and *B. anthracis* [15].

**Figure 6:**
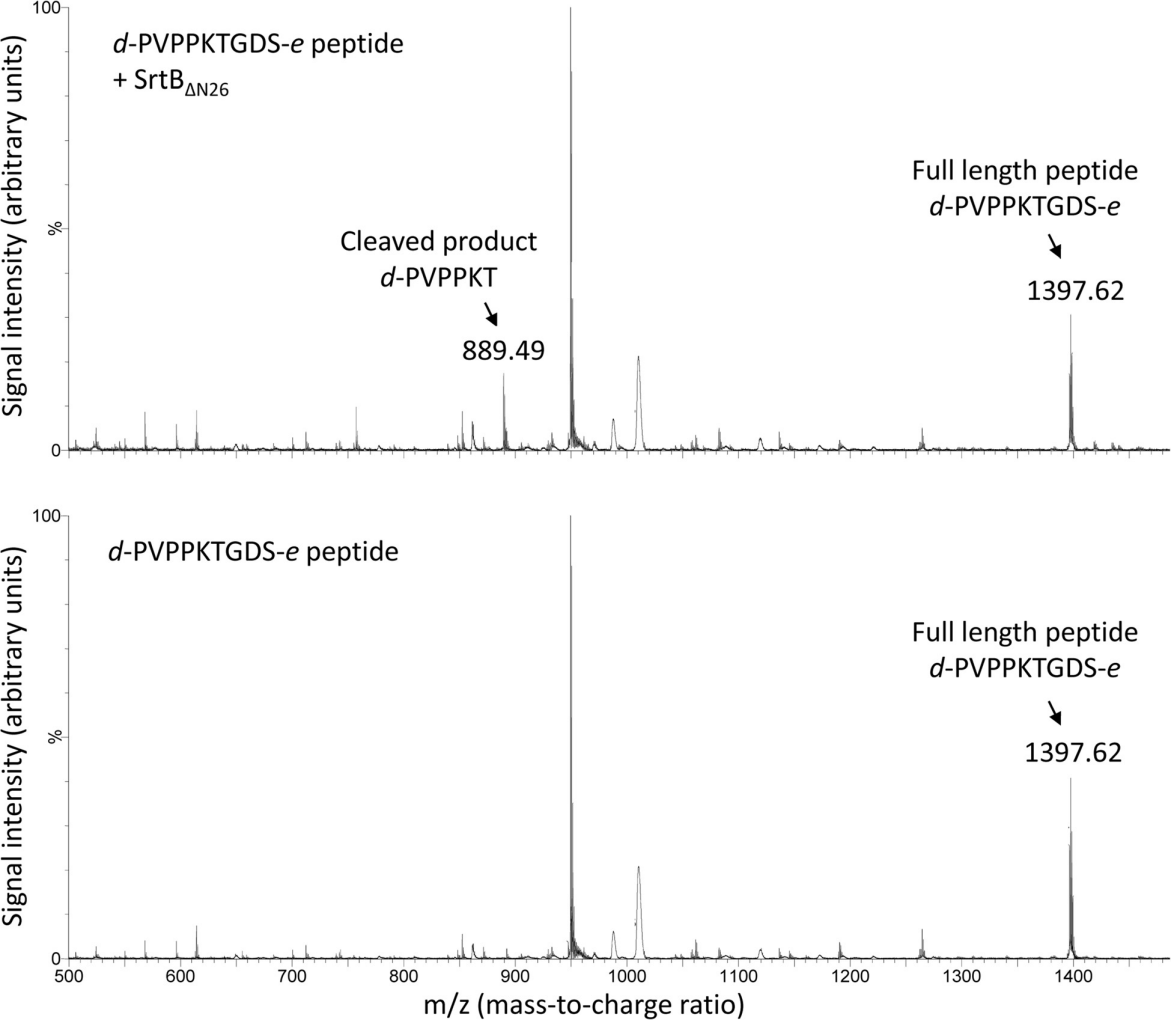
**SrtB**_Δ**N26**_ **cleaves FRET peptides between T and G residues.** MALDI analysis of FRET reaction products revealed a fragment of mass 889.46, corresponding to the predicted mass of *d-*PVPPKT-OH (top) when *d-*PVPPKTGDS-*e* was incubated with SrtB_ΔN26_. This fragment was absent in the mock treated peptide sample (bottom), indicating that SrtB_ΔN26_ cleaves the *d-*PVPPKTGDS*-e* between the T and G residues.

The cleavage of the SPKTG, PPKTG, and SPQTG motifs was enhanced at least two-fold by the addition of the two native amino acids immediately downstream of this sequon (data not shown). Analysis of the FRET reaction with these modified peptides revealed that SrtB_ΔN26,_ cleaves these peptides between the T and G residues. MALDI analysis of *d*-PVPPKTGDS-*e* peptide incubated with SrtB_ΔN26_ results in a peptide with a mass of 889 Da, corresponding to the fragment *d*-PVPPKT-OH (Figure 6, top). The peptide control, incubated without SrtB_ΔN26,_ lacked this fragment (Figure 6, bottom). Cleavage between the T and G residues for the *d*-SDSPKTGDN-*e* and *d*-IHSPQTGDV-*e* peptides was also confirmed (data not shown), indicating that *C. difficile* SrtB cleaves the (S/P)PXTG motif between the same residues as other functional sortases [4,15,38,39].

### Kinetic measurements of SrtB activity

**Figure 7:**
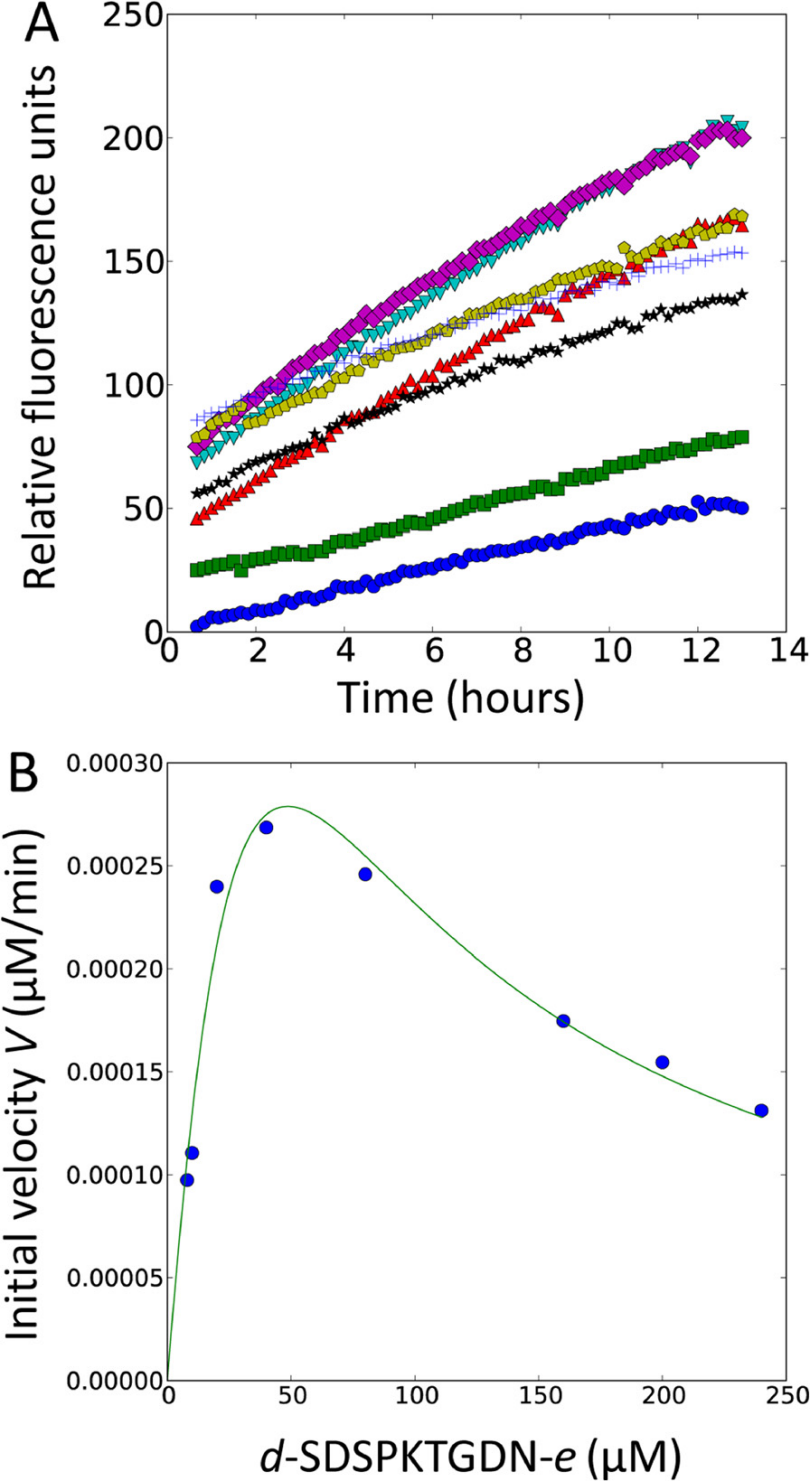
**Kinetic parameters of SrtB**_Δ**N26**_ **.** In order to determine the *in vitro* kinetic parameters of SrtB_ΔN26_ for the SPKTG and PPKTG motifs, we performed a kinetic analysis of the sortase-catalyzed hydrolysis reaction. **A**. Progress curves of the SrtB_ΔN26_-catalyzed hydrolysis reactions at various concentrations of *d-*SDSPKTGDN*-e* [8 (blue ●), 10 (green ▪), 20 (red ▲), 40 (teal ▼), 80 (purple ♦), 160 (yellow ), 200 (black ★), and 240 μM (blue **+**). The steady state rate (*V*) was determined by fitting the data to a linear function. **B**. Plot of *V* against the concentration of the peptide [*S*]. Nonlinear regression of these data fitted to Equation 1 resulted in a *K*_*m*_ of 74.7 ± 48.2 μM for *d-*SDSPKTGDN*-e*. SrtB_ΔN26_ is subject to substrate inhibition at peptide concentrations > 30 μM, which is not expected to be physiologically relevant.

In order to calculate the *in vitro* kinetic parameters of SrtB_ΔN26_ for the *d*-SDSPKTGDN-*e* and *d*-PVPPKTGDS-*e* peptides, we performed a kinetic analysis of the sortase-catalyzed hydrolysis reaction. Figure 7A shows the progress curves of the SrtB_ΔN26_ catalyzed hydrolysis reactions at various *d-*SDSPKTGDN*-e* concentrations. For each progress curve, the amount of fluorescent product (after conversion from RFU to concentration) was approximately 5% of the initial substrate concentration. Within the time period analyzed, the progress curves are linear, so the steady state rate (*V*) was determined by fitting the data to a linear function. Figure 7B shows *V* plotted against the concentration of the peptide. Non-linear regression of these data fitted to a modified Michaelis-Menten equation incorporating substrate inhibition (Equation 1):

1 $$ V=\frac{V_{max}\cdot \left[S\right]}{K_m+\left[S\right]+\frac{{\left[S\right]}^2}{K_i}} $$

Using SciPy 0.11.0 in Python 2.7.3, where *V*_*max*_ is the apparent maximal enzymatic velocity, *K*_*m*_ is the apparent Michaelis constant, and *K*_*i*_ is the apparent inhibitor dissociation constant for unproductive substrate binding. This resulted in a *K*_*m*_ of 74.7 ± 48.2 μM and a *K*_*cat*_ of 1.1×10^−3^ ± 6×10^−4^ min^−1^ for *d-*SDSPKTGDN*-e* (Figure 7B). This analysis was performed for *d-*PVPPKTGDS*-e*, resulting in a *K*_*m*_ of 53.3 ± 25.6 μM and a *K*_*cat*_ of 8.3×10^−4^ ± 3×10^−4^ min^−1^. SrtB_ΔN26_ is subject to substrate inhibition; at peptide concentrations greater than 30 μM, the rate of SrtB_ΔN26_ activity decreases. Substrate inhibition has previously been observed for other sortase enzymes *in vitro*, and is not expected to be physiologically relevant [40].

### Inhibiting SrtB activity

**Figure 8:**
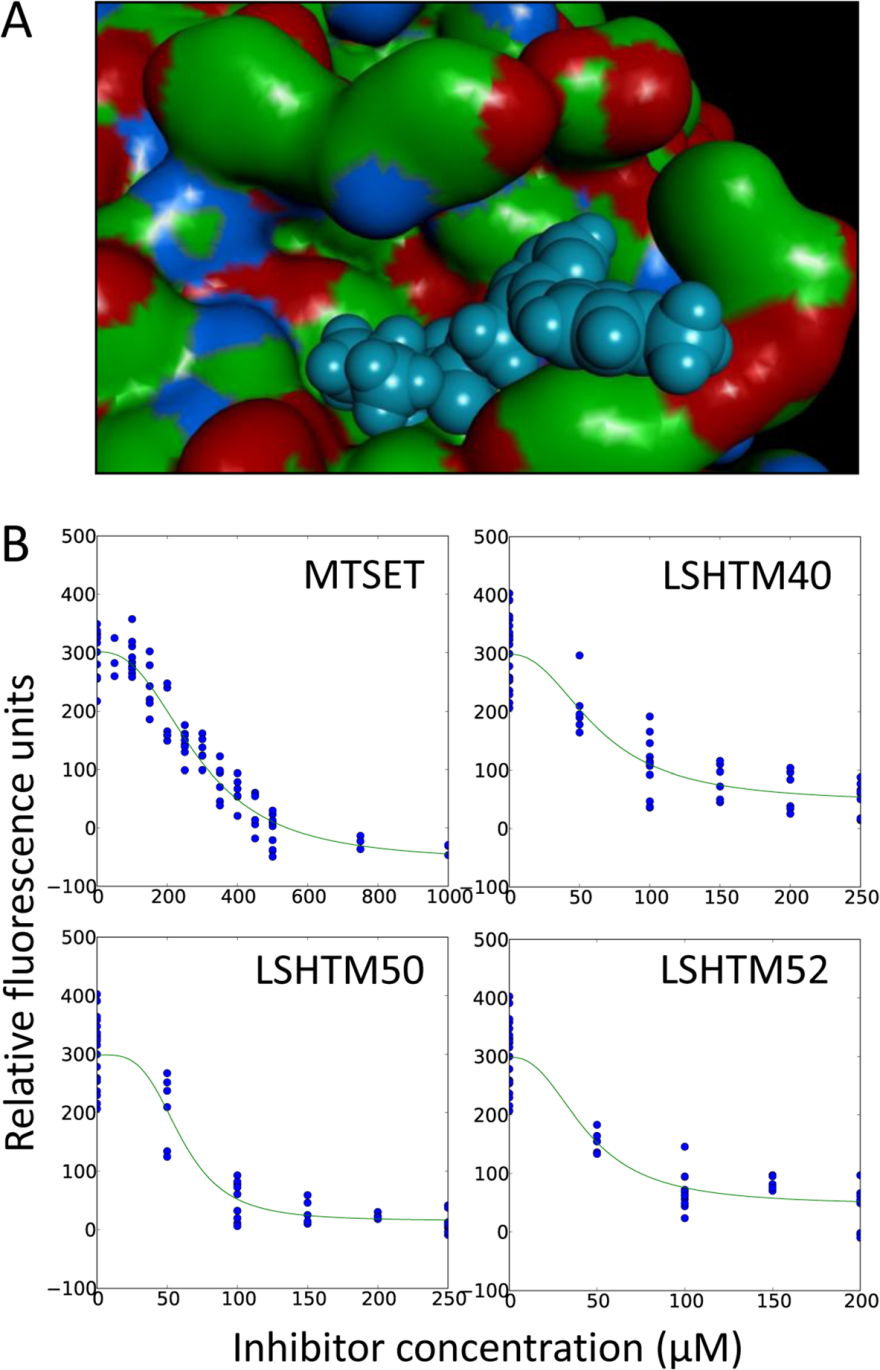
**SrtB**_Δ**N26**_ **activity can be inhibited by rationally designed inhibitors.** The proprietary *LeadBuilder* virtual-screening method (Domainex Ltd) was used to screen a database of 80,000 potential protease inhibitors, *PROTOCATS*, with pharmacophoric and docking filters derived from analysis of the BaSrtB crystal structure [28]. **A**. Space filling model showing one of the hit compounds fitting into the active site of BaSrtB and interacting with the catalytic cysteine residue. **B**. MTSET and the hits from the virtual screen were tested in the FRET-based assay at varying concentrations to screen for inhibition of SrtB_ΔN26_ mediated cleavage of *d-*PVPPKTGDS*-e*. The most effective compounds were LSHTM40, LSHTM50, and LSHTM52, which had IC50 values of 63.1 ± 8.8, 60.1 ± 4.7 and 44.1 ± 6.9 μM, respectively. The IC50 for MTSET was 286.7 ± 16.6 μM, indicating its inhibitory effect on SrtB_ΔN26_ is less potent than the three identified compounds.

We sought to determine whether *C. difficile* SrtB could be specifically targeted using small-molecule inhibitors. The published crystal structure of the *B. anthracis* SrtB (BaSrtB) [28] was used as a template for the selection of potential *C. difficile* SrtB inhibitors. These orthologous proteins show 70% identity and 90% similarity at the active site, and their differences are confined to the periphery of the active site. The proprietary *LeadBuilder* virtual-screening method (Domainex Ltd) was used to interrogate the *PROTOCATS* database of potential protease inhibitors with pharmacophoric and docking filters derived from analysis of the BaSrtB crystal structure. *PROTOCATS* comprises 80,000 commercially-available compounds that may form reversible transition-state-like complexes with protease enzymes. Compounds in *PROTOCATS* contain a carbonyl group which is activated to make a fully reversible complex with the active-site serine/cysteine group by virtue of adjacent moderately electron-withdrawing substituents, which are not leaving groups. Some examples of these functional groups are α-ketoamides and aryl ketones. Figure 8A shows one of the identified compounds docking within the active site structure of BaSrtB.

Compounds identified in this screen as potential SrtB inhibitors were tested alongside the cysteine protease inhibitor MTSET at a range of concentrations in the FRET-based assay using the *d*-PVPPKTGDS-*e* peptide to compare IC50 values. Addition of MTSET reduced SrtB_ΔN26_ activity to below the limits of detection at concentrations of 500 μM and greater. MTSET exhibited an IC50 of 286.7 ± 16.6 μM (Figure 8B). A panel of potential *C. difficile* SrtB inhibitors were screened for inhibition of SrtB_ΔN26_ activity. The most effective of the 62 compounds were LSHTM40, LSHTM50, and LSHTM52. They had IC50 values below 100 μM (Figure 8B, Table 3), at 63.1 ± 8.8 μM, 60.1 ± 4.7 μM, and 44.1 ± 6.9 μM, respectively, showing a good efficacy against *C. difficile* SrtB activity.

**Table 3:** **Structure of most effective inhibitors of SrtB**_Δ**N26**_

**Compound**	**Structure**	**IC50**
LSHTM-0040		63.1 ± 8.8 μM
LSHTM-0050		60.1 ± 4.7 μM
LSHTM-0052		44.1 ± 6.9 μM

## Discussion

*C. difficile* infection is invariably associated with the disruption of the normal intestinal microflora by the administration of broad spectrum antibiotics. Thus there is a pressing need to develop therapies that selectively target *C. difficile* while leaving the intestinal microflora intact. The *C. difficile* reference strain 630 encodes a single predicted sortase, CD630_27180, which has strong amino acid similarity with SrtB of *S. aureus* and *B. anthracis* [24]. Sortase substrates frequently contribute toward pathogenesis via their involvement in attachment to specific tissues during infection [17,41–44], as well as the bacteria’s ability to evade the immune response of the host [32,36]. Sortases, although not essential for growth or viability of the organism, are often essential for virulence in Gram-positive organisms; inactivation of sortases reduces colonization in mice [8,13,44,45], and decreases adhesion and invasion *in vitro* [8,10,14,46,47]. Sortases and their substrates are considered promising targets for the development of new anti-infective compounds [10,14,48]. Unusually for Gram-positive bacteria, *C. difficile* appears to possess a single sortase enzyme that is likely to be important for the viability of the pathogen as we have been unable to construct a *C. difficile* strain 630 SrtB defined mutant (unpublished data). Inhibiting the *C. difficile* sortase could prove to be a strategy to specifically target *C. difficile*.

In this study, we cloned, expressed and characterized the sortase encoded by *CD630_27180* of *C. difficile* 630, a predicted class B sortase (SrtB). Sortase nomenclature is based on sequence similarity to the known classes of sortase, A-F [7]. Sortases of class B typically are involved in heme-iron uptake and tend to be expressed in operons with their substrates [17,18]. Genes encoding class A sortases are not found in proximity to their substrates, which consist of a variety of surface proteins with diverse biological functions. Several exceptions to these rules have already been described, notably a class B sortase that polymerizes pilin subunits in *S. pyogenes* [49], and a class E sortase from *C. diphtheriae* that serves a housekeeping function [50]. The potential *C. difficile* sortase substrates identified in this paper comprise a diverse range of surface proteins, suggesting that SrtB may serve as a housekeeping sortase in *C. difficile*, a function usually reserved for class A sortases.

These potential sortase substrates in *C. difficile* strain 630 comprise of seven proteins, all containing an (S/P)PXTG motif, that are predicted to be surface localized and are conserved across *C. difficile* strains. Recently it was proposed that a *C. difficile* collagen binding protein, CbpA, may be sorted to the cell surface by sortase recognizing an NVQTG motif [30]. In this study, we developed a FRET-based assay to demonstrate that SrtB of *C. difficile* recognizes and cleaves the (S/P)PXTG motif between the threonine and glycine residues, and that cleavage is dependent on a single cysteine residue at position 209. SrtB_ΔN26_ does not appear to cleave the *S. aureus* SrtA and SrtB motifs, LPXTG and NPQTN, respectively, nor the NVQTG motif *in vitro*, suggesting that CbpA from *C. difficile* may be attached to the cell surface by another mechanism.

The FRET-based assay enabled us to determine kinetic parameters for the recombinant *C. difficile* SrtB. Although the catalytic activity appears low, low catalytic efficiency is observed for most sortases *in vitro* [40,51]. The kinetic and cleavage data we report for SrtB_ΔN26_ is consistent with this trend. *In vivo*, the sorting motifs are part of a larger protein, and the transpeptidation substrates are part of a cell wall precursor or mature peptidoglycan [5,6,39]. The transpeptidation reaction has been observed *in vitro* for sortases from bacteria with a Lys-type peptidoglycan, where cross-linking occurs through a peptide bridge [52,53] such as *S. aureus* and *Streptococcus* species [4,40,54], but not for bacteria with Dap-type peptidoglycan such as *Bacillus* with direct cross-linkages through *m*-diaminopimelic acid [55]. The likely cell wall anchor of the *C. difficile* SrtB substrates is the diaminopimelic acid cross-link [56], similar to *Bacillus*. When transpeptidation is observed *in vitro*, the cleavage efficiency of sortase increases.

This study revealed that recombinant SrtB_ΔN26_ cleaves the (S/P)PXTG motifs with varying levels of efficiency, cleaving the sequences PPKTG and SPQTG with the greatest efficiency. Apparent preferential cleavage efficiency of certain substrate sequences *in vitro* has been observed in other sortases. For example, in *B. anthracis*, BaSrtA cleaves LPXTG peptides more readily than a peptide containing the sequence LPNTA [15]. The biological significance of this peptide sequence preference is unknown.

Small-molecule inhibitors with activity against SrtA and SrtB have been reported that prevent cleavage of fluorescently-labelled peptide compounds by sortase *in vitro* [57]. These compounds inhibit cell adhesion to fibronectin, yet, they have no effect on *in vitro* growth. Inhibitors tested against SrtA, SrtB and SrtC in *B. anthracis* irreversibly modified the active cysteine residue [58]. Several compounds identified in this study had an inhibitory effect on *C. difficile* SrtB activity. However, these lead compounds had no direct effect on *in vitro C. difficile* growth (data not shown), which is consistent with observations in *S. aureus* [57]. Inhibition of bacterial growth is not considered vital in the development of sortase-based drug therapies. In both *Staphylococcus* and *Bacillus*, sortase inhibitors show good suitability for further development as therapeutics despite their lack of bactericidal activity. When mice challenged with *S. aureus* were treated with sortase inhibitor compounds, infection rates and mortality were reduced [59], despite these compounds having no effect on staphylococcal growth [57]. The use of *in silico* approaches such as the LeadBuilder method employed by this study to screen databases of putative small-molecule inhibitors for further analysis has been validated. Further analysis of the structural similarities between the hit compounds could lead to a refinement of SrtB inhibitor design and increased potency *in vitro*.

## Conclusions

In conclusion, we demonstrate that *C. difficile* encodes a single sortase, SrtB, with *in vitro* activity. We have confirmed the *C. difficile* SrtB recognition sequence as (S/P)PXTG, and show that *C. difficile* SrtB cleaves the (S/P)PXTG motif within peptides between the threonine and glycine residues. The cysteine residue within the predicted active site is essential for activity of the enzyme, and the cleavage of fluorescently-labelled peptides can be inhibited by MTSET, a known cysteine protease inhibitor. SrtB inhibitors identified through our *in silico* screen show a greater level of efficacy then MTSET at inhibiting the protease activity of *C. difficile* SrtB. Such inhibitors provide a significant step in successfully identifying *C. difficile* SrtB inhibitor compounds, which can be further refined to enhance their efficacy, and may contribute towards the development of novel selective therapeutics against CDI.

## Methods

### Bacterial culture

*C. difficile* strain 630 [24] was cultured on Brazier’s agar (BioConnections) supplemented with 4% egg yolk (BioConnections) and 1% defibrinated horse blood (TCS Biosciences Ltd.). Liquid cultures were grown in brain heart infusion broth (Oxoid Ltd.) supplemented with 0.05% L-cysteine (BHIS broth). All media was supplemented with *C. difficile* antibiotic supplement (250 μg/ml D-cycloserine and 8 μg/ml cefoxitin, BioConnections). *C. difficile* cultures were incubated at 37°C for 24–48 hours in a Whitley MG500 anaerobic workstation (Don Whitley Scientific Ltd.).

One Shot Top10® (Invitrogen) and XL-1 Blue (Agilent) *Escherichia coli* were used for all cloning steps, and NiCo21(DE3) *E. coli* (NEB) was used for the expression of recombinant proteins [60]. *E. coli* strains were grown at 37°C on Luria-Bertani (LB) agar (Novagen) or in LB broth (Difco). Media was supplemented with 100 μg/ml ampicillin or 50 μg/ml kanamycin as required.

### Genomic DNA isolation

Genomic DNA was isolated from *C. difficile* strain 630 [24,61] by phenol chloroform extraction as previously described [29] and used as a template for cloning. The annotated genome sequences from *C. difficile* strains R20291 and CD196 (RT027) [29], M68 and CF5 (RT017) [20], M120 (RT078) [20], and CD305 (RT023) (unpublished, Wellcome Trust Sanger Institute) were used for analysis.

### Identification of sortase substrates

All proteins encoded by *C. difficile* strain 630 [24,61] were searched for the patterns (S/P)PXTG [11] and NVQTG [30] positioned 17–45 amino acid residues from the C-terminus [31]. The resulting candidate protein list was assessed for the known features of a sortase substrate: (i) a suitable N-terminal signal peptide sequence, as predicted by SignalP 4.1 Server (http://www.cbs.dtu.dk/services/SignalP/) [62], (ii) a potential transmembrane segment following the C-terminal “LPXTG-like” sequence, as predicted by TMHMM Server v. 2.0 (http://www.cbs.dtu.dk/services/TMHMM/) [63], and (iii) at least two consecutive basic residues (arginine or lysine) at the C-terminus [31–33].

### Genetic manipulation

A list of primers and plasmids used in this study can be found in Tables 4 and 5, respectively. The coding sequence for *srtB* (CD630_27180) was codon-optimized for expression in *E. coli* by Celtek Bioscience, LLC (Nashville, TN, USA). The resulting fragment was cloned into the NcoI/XhoI sites of pET28a using primers pET_3 and pET_4 to create pET28a-*srtB*. To improve soluble SrtB yield, the N-terminal transmembrane anchor domain (residues 2–25) was replaced with a six-histidine tag. The truncated gene *srtB*_ΔN26_ was amplified from pET28a-*srtB* using primers pET_17 and pET_16, and cloned into the NcoI/XhoI sites of pET28a to create pET28a-*srtB*_ΔN26_. The mutant protein SrtB_ΔN26,C209A_ was generated using the QuikChange Site-Directed Mutagenesis kit (Agilent) in accordance with the manufacturer’s instructions using pET28a-*srtB*_ΔN26_ as a template and primers C209A and C209A_antisense. Successful construction of recombinant plasmids was confirmed by DNA sequencing using primers T7F and T7R (Source BioScience).

**Table 4:** **Primers used in this study**

**Primer**	**Sequence**
pET_3	gatattccatggatgaagaaactgtaccgtatcgttatc
pET_4	gatgagctcgaggatcagacgaccgtggataacc
pET_17	gatataccatggatgcaccaccaccaccaccactctaaactgaccaaatacaaccacgacac
pET_16	gatgagctcgagttagatcagacgaccgtggataac
C209A	tcgttaccctgtctaccgccacctacgaattcgacg
C209A_antisense	cgtcgaattcgtaggtggcggtagacagggtaacga
T7F	taatacgactcactataggg
T7R	gctagttattgctcagcgg

**Table 5:** **Plasmids used in this study**

**Plasmid**	**Description**	**Reference**
pQE30Xa- *srtB*	Codon optimized *srtB*, synthesized and cloned in pQE30xa	Obtained from Celtek Bioscience, LLC
pET28a	Commercial protein expression vector	Provided by Neil Fairweather
pET28a- *srtB*	Codon optimized *srtB* cloned in pET28a	This work
pET28a- *srtB*_ΔN26_	*srtB* with residues 2–25 replaced with a six-histidine tag	This work
pET28a- *srtB*_ΔN26,C209A_	Same as above, with C209A substitution	This work

### RT-PCR analysis

Total RNA was isolated from *C. difficile* 630 grown in BHIS at early exponential phase (t = 3 hours, OD_600_ = 0.4-0.5), late exponential phase (t = 5 hours, OD_600_ = 0.8-0.9), and stationary phase (t = 24 hours, OD_600_ = 0.6-0.8) using RNAprotect Bacteria Reagent (Qiagen) and the FastRNA Pro Blue Kit (MP Biomedicals LLC., Illkirch, France) in accordance with the manufacturer’s instructions. Genomic DNA was removed from total RNA samples using TURBO DNase (Life Technologies). Equal amounts of RNA were reverse transcribed into complementary DNA (cDNA) for expression analysis. Briefly, one μg random primers (Invitrogen) and 40 units RNasin Plus RNase inhibitor (Promega) was added to one μg RNA in a final volume of 10 μl, and incubated at 65°C for 10 min followed by room temperature for 30 min. The following first-strand mixture was added for cDNA synthesis: four μl of 5x first-strand buffer (Invitrogen), two μl 0.1 M DTT (Invitrogen), two μl 10 mM dNTP mix (New England BioLabs), and 1.5 μl Superscript II (Invitrogen). The reaction mixture was incubated at 25°C for 10 minutes, 42°C for 1 h, and finally 70°C for 15 minutes. RT-PCRs were performed with gene specific primers (Additional file 2: Table S1) using cDNA as a template.

### Purification of recombinant protein

Expression constructs were transformed into *E. coli* NiCo21(DE3) (NEB). Cultures grown at 37°C were induced for expression with 1 mM IPTG when the OD_600_ reached 0.6, and harvested after 5 hours. Cell pellets were resuspended in lysis buffer [1× Bugbuster (Novagen), 50 mM NaH_2_PO_4,_ 300 mM NaCl, 40 mM imidazole, 1 mM DTT, 1 mg/ml lysozyme, and 25 U/ml Benzonase nuclease (Novagen) (pH 7.5)]. Lysates were sonicated on ice for 2 min (15 sec on/off) at 50% Vibra Cell™ high intensity ultrasonic processor (Jencon, Leighton Buzzard, Bedfordshire, UK) before centrifugation at 10,000 rpm for 45 min. The supernatant was passed through a 0.22 μM filter before applying to a 1 ml HisTrap HP column (GE Healthcare), pre-equilibrated with buffer (50 mM NaH_2_PO_4,_ 300 mM NaCl, 40 mM imidazole, 1 mM DTT, pH 7.5). SrtB_ΔN26_ was eluted with an imidazole gradient (40 – 500 mM) over 25 column volumes. Fractions containing SrtB_ΔN26_ (as identified by SDS-PAGE) were pooled and injected onto a HiLoad 16/60 Superdex 200 column (GE Healthcare) pre-equilibrated with buffer F (5 mM CaCl_2_, 50 mM Tris–HCl (pH 7.5), 150 mM NaCl, 1 mM DTT). Eluted fractions containing SrtB_ΔN26_ were pooled and concentrated using an Amicon Ultra-15 (10 kDa) centrifuge filter unit (Millipore). Protein samples were quantified using Bradford reagent (Thermo Scientific) and analyzed by SDS-PAGE. The mutant protein SrtB_ΔN26,C209A_ was expressed and purified following the above method. Expression of SrtB_ΔN26_ and SrtB_ΔN26,C209A_ was confirmed by MALDI fingerprinting.

### Immunoblotting

Samples were resolved on Novex NuPage 10% Bis-Tris SDS-PAGE gels (Invitrogen) before transferring to Hybond*-*C Extra nitrocellulose (GE Healthcare). Membranes were probed with rabbit antiserum directed against 6xHis-tag (1:5000, Abcam), followed by goat anti-rabbit IRDye conjugated secondary antibody (1:7500, LI-COR Biotechnology). Blots were visualized using an Odyssey near-infrared imager (LI-COR Biotechnology).

### *In vitro* analysis of sortase activity

SrtB_ΔN26_ activity was monitored using a fluorescence resonance energy transfer (FRET) assay [58] in buffer F (5 mM CaCl_2_, 50 mM Tris–HCl (pH 7.5), 150 mM NaCl, and 1 mM DTT). Fluorescently self-quenched peptides tagged with 5-((2-Aminoethyl)amino)naphthalene-1-sulfonic acid (Edans) as a fluorophore and 4-([4-(Dimethylamino)phenyl]­azo)­benzoic acid (Dabcyl) as a quencher [35], and containing the predicted sorting signals of SrtB were purchased from Protein Peptide Research Ltd and solubilized in DMSO (Table 2). The FRET-based assay was performed in a final volume of 100 μl buffer F containing 10 μM SrtB_ΔN26_ and 20 μM fluorogenic peptide in clear-bottomed, black polystyrene 384-well plates (Nunc). Plates were incubated for 48 hours at 37°C, during which fluorescence (excitation = 340 nm, emission = 490 nm) was measured using a SpectraMax M3 plate reader (Molecular Devices). Five mM 2-(trimethylamonium)ethylmethanethiosulfonate (MTSET, Affymetrix) was added to the reaction as indicated. Each experiment was performed in triplicate with a minimum of three biological replicates, and the results are presented as the means and the standard error of the data obtained. The two-tailed Student’s T-test was used to analyze the data. MALDI analysis of FRET reaction samples was performed by the Protein and Nucleic Acid Chemistry Facility (University of Cambridge) to determine exact cleavage site within each peptide.

### Kinetic measurements

Kinetic data for SrtB_ΔN26_ were obtained by incubating varying concentrations of peptide (8, 10, 20, 40, 80, 160, 200 and 240 μM) with 10 μM SrtB_ΔN26_. All reactions were performed as described above, with fluorescence monitored every ten minutes over a 13 hour period. To correlate fluorescence signal, expressed as arbitrary relative fluorescence units (RFU), with the concentration of product formed, standard curves of the fluorophore Edans were collected. The linear segment of the fluorophore standard curve generated a conversion ratio of 703.9 RFU/ μM Edans. Initial velocities (*V*) were determined from the progress curves and plotted against substrate concentration [*S*]. The data were fitted to a modified version of the Michaelis-Menten equation incorporating substrate inhibition using SciPy 0.11.0 in Python 2.7.3, where *V*_*max*_ is the maximal enzymatic velocity, *K*_*m*_ is the Michaelis constant, and *K*_*i*_ is the inhibitor dissociation constant for unproductive substrate binding. All data points were collected in triplicate, and the overall assay was run in duplicate.

### Identification of SrtB inhibitors

The proprietary *LeadBuilder* virtual screening method (Domainex, Ltd) was used to interrogate a database (*PROTOCATS*) of 80,000 potential compounds which had been pre-selected as protease inhibitors. The virtual screening protocol used pharmacophoric and docking filters derived from analysis of the BaSrtB crystal structure (with which the *C. difficile* SrtB shows 70% identity and 90% similarity at the active site). Sixty-two compounds identified in this screen as potential SrtB inhibitors were obtained from Enamine, ChemBridge, and Key Organics, and solubilized in DMSO. Selected compounds and MTSET were incubated with 10 μM SrtB_ΔN26_ at a range of concentrations in the FRET-based assay conditions described above, so that final DMSO concentrations were ≤ 3.75%, a concentration shown to have no significant effect on control fluorescence (data not shown). IC50 values were determined by non-linear least squares fit to a four parameter sigmoidal function using SciPy 0.11.0 in Python 2.7.3.

## Additional files

Additional file 1: Figure S1. RT-PCR analysis in *C. difficile* strain 630 of *CD2718* and its predicted substrates. PCR reactions were performed with 630 cDNA that was prepared from cultures grown to early exponential (E), late exponential (L) and stationary phase (S). M = Hyperladder I (Bioline), G = 630 genomic DNA, W = dH_2_O. A “+“indicates cDNA reaction with added reverse transcriptase, “-“ indicates cDNA reaction without added reverse transcriptase (control for DNA depletion of RNA sample). Additional file 2: Table S1. Primers used for RT-PCR analysis.

## Abbreviations

SrtA: Sortase A
SrtB: Sortase B
FRET: Fluorescence resonance energy transfer
MTSET: 2-(trimethylamonium)ethylmethanethiosulfonate
Dabcyl or *d*: 4-([4-(dimethylamino)phenyl]azo)-benzoyl
Edans or *e*: 5-((2-Aminoethyl)amino)naphthalene-1-sulfonic acid
CDI: *C. difficile* infection
IPTG: Isopropyl β-D-1-thiogalactopyranoside
RFU: Relative fluorescence units

## References

[1] Mazmanian SK, Ton-That H, Schneewind O: **Sortase-catalysed anchoring of surface proteins to the cell wall of*****Staphylococcus aureus*****.** *Mol Microbiol* 2001, **40**(5):1049–1057. 10.1046/j.1365-2958.2001.02411.x 11401711

[2] Ton-That H, Faull KF, Schneewind O (1997). Anchor structure of staphylococcal surface proteins. A branched peptide that links the carboxyl terminus of proteins to the cell wall. J Biol Chem.

[3] Ton-That H, Mazmanian SK, Alksne L, Schneewind O: **Anchoring of surface proteins to the cell wall of*****Staphylococcus aureus*****. Cysteine 184 and histidine 120 of sortase form a thiolate-imidazolium ion pair for catalysis.** *J Biol Chem* 2002, **277**(9):7447–7452. 10.1074/jbc.M109945200 11714722

[4] Ton-That H, Mazmanian SK, Faull KF, Schneewind O: **Anchoring of surface proteins to the cell wall of*****Staphylococcus aureus*****. Sortase catalyzed*****in vitro*****transpeptidation reaction using LPXTG peptide and NH(2)-Gly(3) substrates.** *J Biol Chem* 2000, **275**(13):9876–9881. 10.1074/jbc.275.13.9876 10734144

[5] Perry AM, Ton-That H, Mazmanian SK, Schneewind O: **Anchoring of surface proteins to the cell wall of*****Staphylococcus aureus.*****III. Lipid II is an in vivo peptidoglycan substrate for sortase-catalyzed surface protein anchoring.** *J Biol Chem* 2002, **277**(18):16241–16248. 10.1074/jbc.M109194200 11856734

[6] Ruzin A, Severin A, Ritacco F, Tabei K, Singh G, Bradford PA, Siegel MM, Projan SJ, Shlaes DM (2002). Further evidence that a cell wall precursor [C(55)-MurNAc-(peptide)-GlcNAc] serves as an acceptor in a sorting reaction. J Bacteriol.

[7] Spirig T, Weiner EM, Clubb RT (2011). Sortase enzymes in Gram-positive bacteria. Mol Microbiol.

[8] Mazmanian SK, Liu G, Jensen ER, Lenoy E, Schneewind O: ***Staphylococcus aureus*****sortase mutants defective in the display of surface proteins and in the pathogenesis of animal infections.** *Proc Natl Acad Sci U S A* 2000, **97**(10):5510–5515. 10.1073/pnas.080520697 PMC25859 10805806

[9] Mazmanian SK, Liu G, Ton-That H, Schneewind O: ***Staphylococcus aureus*****sortase, an enzyme that anchors surface proteins to the cell wall.** *Science* 1999, **285**(5428):760–763. 10.1126/science.285.5428.760 10427003

[10] Kharat AS, Tomasz A: **Inactivation of the*****srtA*****gene affects localization of surface proteins and decreases adhesion of*****Streptococcus pneumoniae*****to human pharyngeal cells*****in vitro*****.** *Infect Immun* 2003, **71**(5):2758–2765. 10.1128/IAI.71.5.2758-2765.2003 PMC153252 12704150

[11] Pallen MJ, Lam AC, Antonio M, Dunbar K (2001). An embarrassment of sortases - a richness of substrates?. Trends Microbiol.

[12] Barnett TC, Scott JR: **Differential recognition of surface proteins in*****Streptococcus pyogenes*****by two sortase gene homologs.** *J Bacteriol* 2002, **184**(8):2181–2191. 10.1128/JB.184.8.2181-2191.2002 PMC134975 11914350

[13] Bierne H, Mazmanian SK, Trost M, Pucciarelli MG, Liu G, Dehoux P, Jansch L, Garcia-del Portillo F, Schneewind O, Cossart P: **Inactivation of the*****srtA*****gene in*****Listeria monocytogenes*****inhibits anchoring of surface proteins and affects virulence.** *Mol Microbiol* 2002, **43**(4):869–881. 10.1046/j.1365-2958.2002.02798.x 11929538

[14] Garandeau C, Reglier-Poupet H, Dubail I, Beretti JL, Berche P, Charbit A: **The sortase SrtA of*****Listeria monocytogenes*****is involved in processing of internalin and in virulence.** *Infect Immun* 2002, **70**(3):1382–1390. 10.1128/IAI.70.3.1382-1390.2002 PMC127754 11854224

[15] Gaspar AH, Marraffini LA, Glass EM, Debord KL, Ton-That H, Schneewind O: ***Bacillus anthracis*****sortase A (SrtA) anchors LPXTG motif-containing surface proteins to the cell wall envelope.** *J Bacteriol* 2005, **187**(13):4646–4655. 10.1128/JB.187.13.4646-4655.2005 PMC1151759 15968076

[16] Swaminathan A, Mandlik A, Swierczynski A, Gaspar A, Das A, Ton-That H: **Housekeeping sortase facilitates the cell wall anchoring of pilus polymers in*****Corynebacterium diphtheriae*****.** *Mol Microbiol* 2007, **66**(4):961–974. 10.1111/j.1365-2958.2007.05968.x PMC2841690 17919283

[17] Mazmanian SK, Ton-That H, Su K, Schneewind O: **An iron-regulated sortase anchors a class of surface protein during*****Staphylococcus aureus*****pathogenesis.** *Proc Natl Acad Sci U S A* 2002, **99**(4):2293–2298. 10.1073/pnas.032523999 PMC122358 11830639

[18] Maresso AW, Chapa TJ, Schneewind O: **Surface protein IsdC and Sortase B are required for heme-iron scavenging of*****Bacillus anthracis*****.** *J Bacteriol* 2006, **188**(23):8145–8152. 10.1128/JB.01011-06 PMC1698196 17012401

[19] Rupnik M, Wilcox MH, Gerding DN: ***Clostridium difficile*****infection: new developments in epidemiology and pathogenesis.** *Nat Rev Microbiol* 2009, **7**(7):526–536. 10.1038/nrmicro2164 19528959

[20] He M, Sebaihia M, Lawley TD, Stabler RA, Dawson LF, Martin MJ, Holt KE, Seth-Smith HM, Quail MA, Rance R, Brooks K, Churcher C, Harris D, Bentley SD, Burrows C, Clark L, Corton C, Murray V, Rose G, Thurston S, van Tonder A, Walker D, Wren BW, Dougan G, Parkhill J: **Evolutionary dynamics of*****Clostridium difficile*****over short and long time scales.** *Proc Natl Acad Sci U S A* 2010, **107**(16):7527–7532. 10.1073/pnas.0914322107 PMC2867753 20368420

[21] Dingle KE, Griffiths D, Didelot X, Evans J, Vaughan A, Kachrimanidou M, Stoesser N, Jolley KA, Golubchik T, Harding RM, Peto TE, Fawley, Walker AS, Wilcox M, Crook DW: **Clinical*****Clostridium difficile*****: clonality and pathogenicity locus diversity.** *PLoS One* 2011, **6**(5):e19993. 10.1371/journal.pone.0019993 PMC3098275 21625511

[22] Stabler RA, Dawson LF, Valiente E, Cairns MD, Martin MJ, Donahue EH, Riley TV, Songer JG, Kuijper EJ, Dingle KE, Wren BW: **Macro and micro diversity of*****Clostridium difficile*****isolates from diverse sources and geographical locations.** *PLoS One* 2012, **7**(3):e31559. 10.1371/journal.pone.0031559 PMC3292544 22396735

[23] Cleary RK: ***Clostridium difficile*****-associated diarrhea and colitis - Clinical manifestations, diagnosis and treatment.** *Dis Colon Rectum* 1998, **41**(11):1435–1449. 10.1007/BF02237064 9823813

[24] Sebaihia M, Wren BW, Mullany P, Fairweather NF, Minton N, Stabler R, Thomson NR, Roberts AP, Cerdeno-Tarraga AM, Wang H, Holden MT, Wright A, Churcher C, Quail MA, Baker S, Bason N, Brooks K, Chillingworth T, Cronin A, Davis P, Dowd L, Fraser A, Feltwell T, Hance Z, Holroyd S, Jagels K, Moule S, Mungall K, Price C, Rabbinowitsch E, *et al*: **The multidrug-resistant human pathogen*****Clostridium difficile*****has a highly mobile, mosaic genome.** *Nat Genet* 2006, **38**(7):779–786. 10.1038/ng1830 16804543

[25] Liew CK, Smith BT, Pilpa R, Suree N, Ilangovan U, Connolly KM, Jung ME, Clubb RT: **Localization and mutagenesis of the sorting signal binding site on sortase A from*****Staphylococcus aureus*****.** *FEBS Lett* 2004, **571**(1–3):221–226. 10.1016/j.febslet.2004.06.070 15280046

[26] Marraffini LA, Ton-That H, Zong Y, Narayana SV, Schneewind O: **Anchoring of surface proteins to the cell wall of*****Staphylococcus aureus.*****A conserved arginine residue is required for efficient catalysis of sortase A.** *J Biol Chem* 2004, **279**(36):37763–37770. 10.1074/jbc.M405282200 15247224

[27] Kelley LA, Sternberg MJ (2009). Protein structure prediction on the Web: a case study using the Phyre server. Nat Protoc.

[28] Zhang R, Wu R, Joachimiak G, Mazmanian SK, Missiakas DM, Gornicki P, Schneewind O, Joachimiak A: **Structures of sortase B from*****Staphylococcus aureus*****and*****Bacillus anthracis*****reveal catalytic amino acid triad in the active site.** *Structure* 2004, **12**(7):1147–1156. 10.1016/j.str.2004.06.001 PMC2792001 15242591

[29] Stabler RA, He M, Dawson L, Martin M, Valiente E, Corton C, Lawley TD, Sebaihia M, Quail MA, Rose G, Gerding DN, Gibert M, Popoff MR, Parkhill J, Dougan G, Wren BW: **Comparative genome and phenotypic analysis of*****Clostridium difficile*****027 strains provides insight into the evolution of a hypervirulent bacterium.** *Genome Biol* 2009, **10**(9):R102. 10.1186/gb-2009-10-9-r102 PMC2768977 19781061

[30] Tulli L, Marchi S, Petracca R, Shaw HA, Fairweather NF, Scarselli M, Soriani M, Leuzzi R: **CbpA: a novel surface exposed adhesin of*****Clostridium difficile*****targeting human collagen.** *Cell Microbiol* 2013, **15**(10):1674–1687. 10.1111/cmi.12139 23517059

[31] Comfort D, Clubb RT (2004). A comparative genome analysis identifies distinct sorting pathways in gram-positive bacteria. Infect Immun.

[32] Schneewind O, Mihaylova-Petkov D, Model P (1993). Cell wall sorting signals in surface proteins of gram-positive bacteria. EMBO J.

[33] Janulczyk R, Rasmussen M (2001). Improved pattern for genome-based screening identifies novel cell wall-attached proteins in gram-positive bacteria. Infect Immun.

[34] Pritz S, Wolf Y, Kraetke O, Klose J, Bienert M, Beyermann M (2007). Synthesis of biologically active peptide nucleic acid-peptide conjugates by sortase-mediated ligation. J Org Chem.

[35] Matayoshi ED, Wang GT, Krafft GA, Erickson J (1990). Novel fluorogenic substrates for assaying retroviral proteases by resonance energy transfer. Science.

[36] Ton-That H, Liu G, Mazmanian SK, Faull KF, Schneewind O: **Purification and characterization of sortase, the transpeptidase that cleaves surface proteins of*****Staphylococcus aureus*****at the LPXTG motif.** *Proc Natl Acad Sci U S A* 1999, **96**(22):12424–12429. 10.1073/pnas.96.22.12424 PMC22937 10535938

[37] Ton-That H, Schneewind O (1999). Anchor structure of staphylococcal surface proteins. IV. Inhibitors of the cell wall sorting reaction. J Biol Chem.

[38] Dhar G, Faull KF, Schneewind O: **Anchor structure of cell wall surface proteins in*****Listeria monocytogenes*****.** *Biochemistry (Mosc)* 2000, **39**(13):3725–3733. 10.1021/bi992347o 10736172

[39] Marraffini LA, Schneewind O (2005). Anchor structure of staphylococcal surface proteins. V. Anchor structure of the sortase B substrate IsdC. J Biol Chem.

[40] Race PR, Bentley ML, Melvin JA, Crow A, Hughes RK, Smith WD, Sessions RB, Kehoe MA, McCafferty DG, Banfield MJ: **Crystal structure of*****Streptococcus pyogenes*****sortase A: implications for sortase mechanism.** *J Biol Chem* 2009, **284**(11):6924–6933. 10.1074/jbc.M805406200 PMC2652338 19129180

[41] McDevitt D, Francois P, Vaudaux P, Foster TJ: **Molecular characterization of the clumping factor (fibrinogen receptor) of*****Staphylococcus aureus*****.** *Mol Microbiol* 1994, **11**(2):237–248. 10.1111/j.1365-2958.1994.tb00304.x 8170386

[42] Ni Eidhin D, Perkins S, Francois P, Vaudaux P, Hook M, Foster TJ: **Clumping factor B (ClfB), a new surface-located fibrinogen-binding adhesin of*****Staphylococcus aureus*****.** *Mol Microbiol* 1998, **30**(2):245–257. 10.1046/j.1365-2958.1998.01050.x 9791170

[43] Patti JM, Jonsson H, Guss B, Switalski LM, Wiberg K, Lindberg M, Hook M: **Molecular characterization and expression of a gene encoding a*****Staphylococcus aureus*****collagen adhesin.** *J Biol Chem* 1992, **267**(7):4766–4772. 1311320

[44] Cheng AG, Kim HK, Burts ML, Krausz T, Schneewind O, Missiakas DM: **Genetic requirements for*****Staphylococcus aureus*****abscess formation and persistence in host tissues.** *FASEB J* 2009, **23**(10):3393–3404. 10.1096/fj.09-135467 PMC2747682 19525403

[45] Weiss WJ, Lenoy E, Murphy T, Tardio L, Burgio P, Projan SJ, Schneewind O, Alksne L: **Effect of*****srtA*****and*****srtB*****gene expression on the virulence of*****Staphylococcus aureus*****in animal models of infection.** *J Antimicrob Chemother* 2004, **53**(3):480–486. 10.1093/jac/dkh078 14762051

[46] Bolken TC, Franke CA, Jones KF, Zeller GO, Jones CH, Dutton EK, Hruby DE: **Inactivation of the*****srtA*****gene in*****Streptococcus gordonii*****inhibits cell wall anchoring of surface proteins and decreases*****in vitro*****and*****in vivo*****adhesion.** *Infect Immun* 2001, **69**(1):75–80. 10.1128/IAI.69.1.75-80.2001 PMC97857 11119491

[47] Mandlik A, Swierczynski A, Das A, Ton-That H: ***Corynebacterium diphtheriae*****employs specific minor pilins to target human pharyngeal epithelial cells.** *Mol Microbiol* 2007, **64**(1):111–124. 10.1111/j.1365-2958.2007.05630.x PMC2844904 17376076

[48] Jonsson IM, Mazmanian SK, Schneewind O, Bremell T, Tarkowski A: **The role of*****Staphylococcus aureus*****sortase A and sortase B in murine arthritis.** *Microbes Infect* 2003, **5**(9):775–780. 10.1016/s1286-4579(03)00143-6 12850203

[49] Kang HJ, Coulibaly F, Proft T, Baker EN: **Crystal structure of Spy0129, a*****Streptococcus pyogenes*****class B sortase involved in pilus assembly.** *PLoS One* 2011, **6**(1):e15969. 10.1371/journal.pone.0015969 PMC3019223 21264317

[50] Chang C, Mandlik A, Das A, Ton-That H: **Cell surface display of minor pilin adhesins in the form of a simple heterodimeric assembly in*****Corynebacterium diphtheriae*****.** *Mol Microbiol* 2011, **79**(5):1236–1247. 10.1111/j.1365-2958.2010.07515.x PMC3043122 21205008

[51] Frankel BA, Kruger RG, Robinson DE, Kelleher NL, McCafferty DG: ***Staphylococcus aureus*****sortase transpeptidase SrtA: insight into the kinetic mechanism and evidence for a reverse protonation catalytic mechanism.** *Biochemistry (Mosc)* 2005, **44**(33):11188–11200. 10.1021/bi050141j 16101303

[52] Dziarski R (2004). Peptidoglycan recognition proteins (PGRPs). Mol Immunol.

[53] Schleifer KH, Kandler O (1972). Peptidoglycan types of bacterial cell walls and their taxonomic implications. Bacteriol Rev.

[54] Necchi F, Nardi-Dei V, Biagini M, Assfalg M, Nuccitelli A, Cozzi R, Norais N, Telford JL, Rinaudo CD, Grandi G, Maione D (2011). Sortase A substrate specificity in GBS pilus 2a cell wall anchoring. PLoS One.

[55] Weiner EM, Robson S, Marohn M, Clubb RT: **The Sortase A enzyme that attaches proteins to the cell wall of*****Bacillus anthracis*****contains an unusual active site architecture.** *J Biol Chem* 2010, **285**(30):23433–23443. 10.1074/jbc.M110.135434 PMC2906334 20489200

[56] Peltier J, Courtin P, El Meouche I, Lemee L, Chapot-Chartier MP, Pons JL: ***Clostridium difficile*****has an original peptidoglycan structure with a high level of N-acetylglucosamine deacetylation and mainly 3–3 cross-links.** *J Biol Chem* 2011, **286**(33):29053–29062. 10.1074/jbc.M111.259150 PMC3190713 21685382

[57] Oh KB, Oh MN, Kim JG, Shin DS, Shin J: **Inhibition of sortase-mediated*****Staphylococcus aureus*****adhesion to fibronectin via fibronectin-binding protein by sortase inhibitors.** *Appl Environ Microbiol* 2006, **70**(1):102–106. 10.1007/s00253-005-0040-8 16010573

[58] Maresso AW, Wu R, Kern JW, Zhang R, Janik D, Missiakas DM, Duban ME, Joachimiak A, Schneewind O (2007). Activation of inhibitors by sortase triggers irreversible modification of the active site. J Biol Chem.

[59] Oh K-B, Nam K-W, Ahn H, Shin J, Kim S, Mar W: **Therapeutic effect of (Z)-3-(2,5-dimethoxyphenyl)-2-(4-methoxyphenyl) acrylonitrile (DMMA) against*****Staphylococcus aureus*****infection in a murine model.** *Biochem Biophys Res Commun* 2010, **396**(2):440–444. 10.1016/j.bbrc.2010.04.113 20433810

[60] Robichon C, Luo J, Causey TB, Benner JS, Samuelson JC: **Engineering*****Escherichia coli*****BL21(DE3) derivative strains to minimize*****E. coli*****protein contamination after purification by immobilized metal affinity chromatography.** *Appl Environ Microbiol* 2011, **77**(13):4634–4646. 10.1128/AEM.00119-11 PMC3127686 21602383

[61] Monot M, Boursaux-Eude C, Thibonnier M, Vallenet D, Moszer I, Medigue C, Martin-Verstraete I, Dupuy B: **Reannotation of the genome sequence of*****Clostridium difficile*****strain 630.** *J Med Microbiol* 2011, **60**(Pt 8):1193–1199. 10.1099/jmm.0.030452-0 21349987

[62] Petersen TN, Brunak S, von Heijne G, Nielsen H (2011). SignalP 4.0: discriminating signal peptides from transmembrane regions. Nat Methods.

[63] Moller S, Croning MD, Apweiler R (2001). Evaluation of methods for the prediction of membrane spanning regions. Bioinformatics.

