# Supplementary figures and images for: Clostridium difficile has a single sortase, SrtB, that can be inhibited by small-molecule inhibitors
Source: BMC Microbiol. 2014 Aug 31;14:219. doi: 10.1186/s12866-014-0219-1 (PMC4155245; doi:10.1186/s12866-014-0219-1)

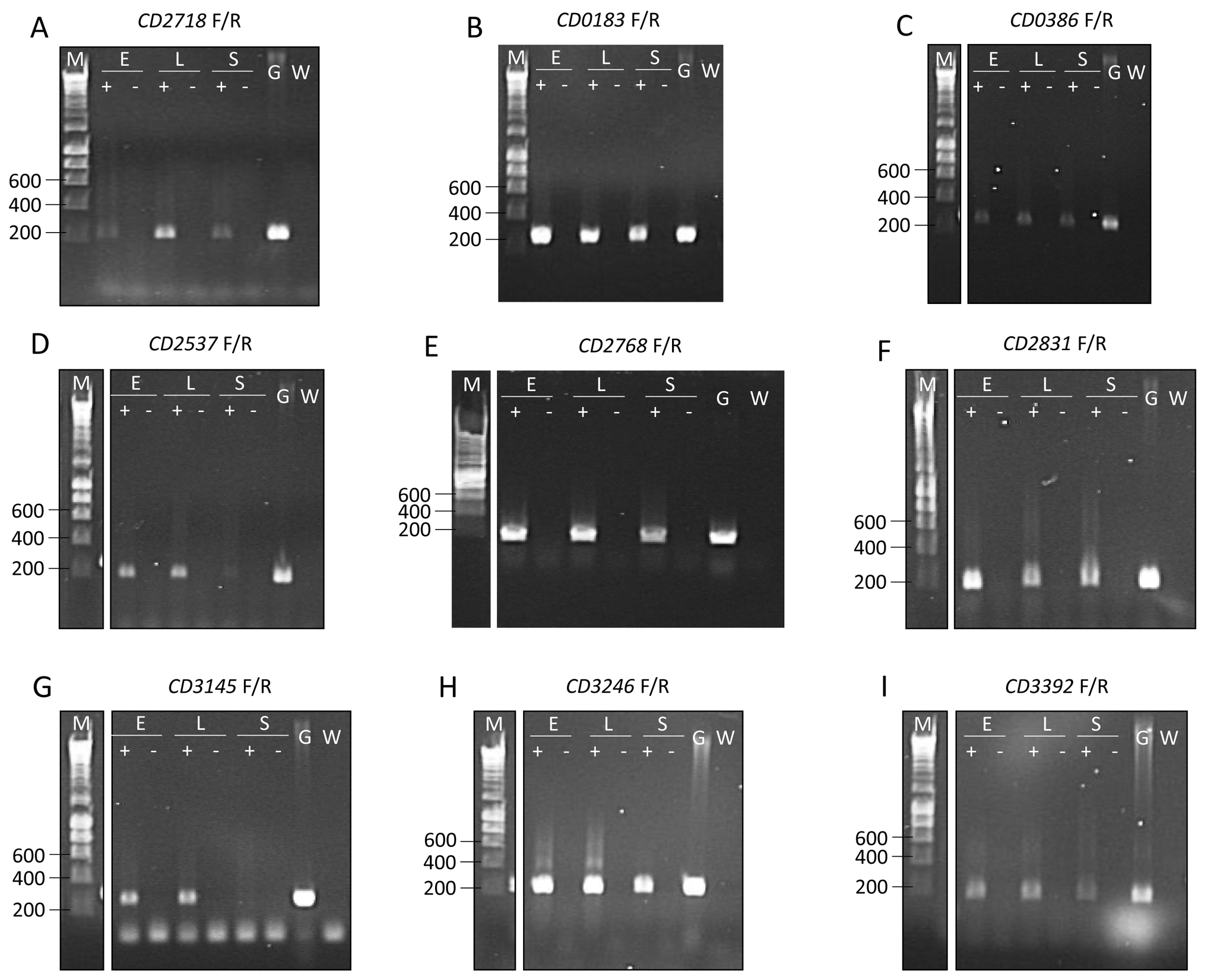

Supplement: Additional file 1: Figure S1. — RT-PCR analysis in C. difficile strain 630 of CD2718 and its predicted substrates. PCR reactions were performed with 630 cDNA that was prepared from cultures grown to early exponential (E), late exponential (L) and stationary phase (S). M = Hyperladder I (Bioline), G = 630 genomic DNA, W = dH2O. A “+“indicates cDNA reaction with added reverse transcriptase, “-“ indicates cDNA reaction without added reverse transcriptase (control for DNA depletion of RNA sample). [file 12866_2014_219_MOESM1_ESM.tiff]
