# Supplementary material for: Clostridium difficile has a single sortase, SrtB, that can be inhibited by small-molecule inhibitors
Source: BMC Microbiol. 2014 Aug 31;14:219. doi: 10.1186/s12866-014-0219-1 (PMC4155245; doi:10.1186/s12866-014-0219-1)
Supplement: Additional file 2: Table S1. — Primers used for RT-PCR analysis. [file 12866_2014_219_MOESM2_ESM.doc]

**Additional file 2: Table S1:** Primers used for RT-PCR analysis

| **Primer** | **Sequence** |
| --- | --- |
| *CD2718* F | AAATTTTGGCTTTCTGTTGAGA |
| *CD2718* R | AGGTTGTTGAACATAGTTTTATTTTTC |
| *CD0183* F | AAAGATGGGGAAACAGAAGGA |
| *CD0183* R | CCCCAAACATATGGACAACC |
| *CD0386* F | GGAGATATTGAGGGCGTGAA |
| *CD0386* R | TTTGCTGTCGTCTGGTTCAG |
| *CD2537* F | CTTTTTGCAAGTGGGAGGAA |
| *CD2537* R | TGGCTCTTTTGAAAAACAAGG |
| *CD2768* F | TAAATGGGGAGCAACTGGAC |
| *CD2768* R | TGAACCACCTGAACCAAAGA |
| *CD2831* F | AAAAGAAGGGGAACCTGTGC |
| *CD2831* R | GCTTCAAAAGCATAACCTTGC |
| *CD3145* F | ATAGGTGGAGGCGGTAATGG |
| *CD3145* R | ACTACCATGTGCACCTCCAT |
| *CD3246* F | AGCAAGCTCAAAAGACGACA |
| *CD3246* R | TCCATCAAATTCAGAACCTGCA |
| *CD3392* F | TATCCATACCACAGCGACCA |
| *CD3392* R | TGATTAGCTCGGCGTTTTCT |
